# Sirt3 restricts tumor initiation via promoting LONP1 deacetylation and K63 ubiquitination

**DOI:** 10.1186/s12967-023-03925-x

**Published:** 2023-02-04

**Authors:** Liyi Wu, Xinyi Yan, Ruibo Sun, Ye Ma, Wanyu Yao, Baogui Gao, Qingyuan Zhang, Junxiong You, Hao Wang, Qinrui Han, Xuegang Sun

**Affiliations:** 1 The Key Laboratory of Molecular Biology, State Administration of Traditional Chinese Medicine, School of Traditional Chinese Medicine, Southern Medical University, Guangzhou, 510515 Guangdong China; 2 Department of Traditional Chinese Medicine, Zhujing Hospital, Southern Medical University, Guangzhou, 510260 Guangdong China

**Keywords:** Sirt3, LONP1, Deacetylation, Ubiquitination, Oncogenesis, Energy metabolism

## Abstract

**Background:**

Sirtuin 3 (Sirt3) is a controversial regulator of carcinogenesis. It residents in the mitochondria and gradually decays during aging. In this study, we tried to investigate the role of Sirt3 in carcinogenesis and to explore its involvement in metabolic alteration.

**Methods:**

We generated conditional intestinal epithelium Sirt3-knockout mice by crossing *Apc*^*Min/*+^; *Villin-Cre* with *Sirt3*^*fl/fl*^ (AVS) mice. The deacetylation site of Lon protease-1 (LONP1) was identified with Mass spectrometry. The metabolic flux phenotype was determined by Seahorse bioanalyzer.

**Results:**

We found that intestinal epithelial cell-specific ablation of Sirt3 promotes primary tumor growth via stabilizing mitochondrial LONP1. Notably, we newly identified that Sirt3 deacetylates human oncogene LONP1 at N terminal residue lysine 145 (K145). The LONP1 hyperacetylation-mutant K145Q enhances oxidative phosphorylation to accelerate tumor growth, whereas the deacetylation-mutant K145R produces calorie-restriction like phenotype to restrain tumorigenesis. Sirt3 deacetylates LONP1 at K145 and subsequently facilitates the ESCRT0 complex sorting and K63-ubiquitination that resulted in the degradation of LONP1. Our results sustain the notion that Sirt3 is a tumor-suppressor to maintain the appropriate ubiquitination and degradation of oncogene LONP1.

**Conclusion:**

Sirt3 represents a targetable metabolic checkpoint of oncogenesis, which produces energy restriction effects via maintaining LONP1 K145 deacetylation and subsequent K63 ubiquitination.

**Supplementary Information:**

The online version contains supplementary material available at 10.1186/s12967-023-03925-x.

## Introduction

Aging is the most potent risk factor for tumorigenesis. Around 70% of cancers occur in population aged 65 years or older [1]. The overall cancer mortality maintains a low level before 45 years and progresses rapidly thereafter, which reaches the peak at about 85 years old [1]. Apart from accumulation of genomic mutations in the process of geroncogenesis, metabolic reprogramming has been supposed to be both the cause and a hallmark of cancer [2]. The natural decline of oxidative phosphorylation and the concomitant shift to Warburg-like metabolic state may act as one of the hits required to drive carcinogenesis.

Sirtuins are a class of nicotinamide adenine dinucleotide (NAD^+^)-dependent deacetylases that are evolutionarily conserved from bacteria to humans. Sirt3 reacts with its required cofactor NAD^+^ to deacetylate mitochondrial protein substrates by generating byproducts such as nicotinamide (NAM) and O-acetyl ADP ribose. So, Sirt3 maintains metabolic adaptations by modulating the global protein acetylation landscape in the mitochondria [3, 4]. Sirt3 also restricts reactive oxygen species (ROS) to a low steady-state level to coordinate metabolic and genetic processes by limiting abnormally high levels of ROS. Expression of Sirt3 in muscle fibers reduces to about 50% in low-functioning elderly participants as compared to the corresponding young individuals [5]. Similarly, aging results in the loss of mitochondrial respiration and a concurrent decline of cytochrome c oxidase activity. Further, the loss of Sirt3 accelerates the development age-related disorders, such as neurodegenerative diseases, cardiovascular diseases, and cancers [6]. Sirt3 has emerged as an indispensable aging suppressor to delay senescence by maintaining mitochondrial homeostasis.

The gradual loss of Sirt3 represents a characteristic of aging which is the most crucial risk factor for carcinogenesis [1, 7]. However, the role of Sirt3 in cancer initiation and progression is still controversial. Sirt3 promotes colorectal carcinogenesis by deacetylating serine hydroxymethyl-transferase 2 (SHMT2) and blocking the degradation of the latter via ubiquitin-lysosome pathway [8]. In line with its role as an oncogene, Sirt3 depletion regresses B cell lymphomagenesis via impairing glutamine flux to the TCA cycle [9]. Paradoxically, Sirt3 loss stabilizes hypoxia-inducible factor-1 α (HIF-1α) and enhances ROS production [10, 11]. Sirt3 overexpression suppresses breast cancer cell proliferation by regressing glycolysis [11]. Similarly, Sirt3 deficiency accelerates the acetylation-dependent deactivation of succinate dehydrogenase complex subunit A to increase H3K4me3 level, which leads to tumour-specific gene transcription [12]. Thus Sirt3 plays dual roles in carcinogenesis through deacetylating different target proteins. In this study, we explored the interaction between Sirt3 and LONP1 and the effect of LONP1 acetylation status on tumorigenesis. Results of this study reveal a new mechanism of Sirt3 in restraining carcinogenesis through a Sirt3-mediated metabolic reprogramming pathway.

## Materials and methods

### Cell culture and transfection

All cell lines, including SW480, AGS, Hela, HEK293T, and U87 cells were obtained from Cell Culture Center of the Chinese Academy of Medical Sciences and were cultured in RPMI-1640 (Gbico) or DMEM (Gbico) containing 10% fetal bovine serum and 1% penicillin/streptomycin at 37 ºC with 5% CO_2_.

SW480 cells were seeded in 24-well plate. Four group of lenti-virus (vector, LONP1-WT, LONP-K145Q and LONP1-K145R) were added into the serum, respectively. stable-expression cell lines were screened with 10 μg/ml Puromycin. Plasmid transfection was conducted using Lipofectamine 2000 according to manufacture’s protocol.

### Immunohistochemistry staining

Tissues were prepared by paraffin after fixation for 48 in 4% paraformaldehyde and cut into 4 μm thickness. The sample was dewaxed by graded ethanol and dimethylbenzene. Antigen retrieval was performed in EDTA buffer routinely. Endogenous peroxidase was inactivated by methanol containing 25% H_2_O_2_. The sections were incubated by primary antibodies respectively (COX2, PCNA, LONP1, Sirt3) overnight in 4 ºC. The second day, the sections were incubated by secondary antibodies after washed by phosphate buffer saline (PBS) for 3 times. Then the slices were counterstained with hematoxylin according to manufacture. The positive area was measured by Image J software [13].

### Immunoprecipitation and immunoblotting analysis

Cells were lysed by modified buffers and immunoprecipitated by corresponding antibody for 12 h at 4 ºC. The A/G beads were added into the contraction according to manufacture and rotated at 4 ºC for 3 h. The A/G beads were boiled in 98 ºC and centrifuged before the protein was separated by SDS-PAGE and transferred onto PVDF membrane for western-blot analysis [14].

### Mitochondria and cytoplasmic fraction extraction

The cells were harvested by trypsin containing 0.25% EDTA and washed by PBS. After centrifuged at 2000 rpm for 5 min, the debris was lysed modified buffer and followed by centrifuge at 800 g for 5 min at 4 ºC to discard cell pullet. The supernatant was collected and then was centrifuged at 15000 g for 10 min at 4 ºC after adding onto separated by 12% SDS-PAGE.

### Immunofluorescence

Mitochondrias were marked by Mito-Tracker according to manufacturer. After washed by PBS, the plate was fixed by 4% paraformaldehyde containing 0.05% Triton-X. Every glass was incubated by 5% BSA diluted in PBS. They were incubated by primary antibodies (LONP1, Sirt3) overnight in 4 ºC. The second day, they were incubated by secondary antibodies [Alexa Fluor 488-labeled Goat Anti-Rabbit IgG(H + L), AMCA-labeled Goat Anti-Mouse IgG(H + L)] and Dapi according to manufacturer. The photograph was taken by confocal microscopy [15].

### Mass spectrum analysis

Mass spectrometry analysis were carried out at Shenzhen Wininnovate Bio Technology Co., Ltd using ThermoFisher Q Exactive mass spectrometer. Information of LONP1 was downloaded from Uniprot (www.uniprot.org/uniprot/P36776).

### Cell proliferation and viability

About 5000 cells of per group were seeded in 96-well plate maintained in complete medium. After adhering for 48 h, cells were stained with Cell Counting Kit-8, and then cell viability was analyzed by microplate reader according to manufacturer protocol [16].

### Cell migration assay

Cells were collected, centrifuged and resuspended with culture medium without FBS. About 5 × 10^4^ cells were seeded in the upper chamber and 600 μl medium with 10% FBS was added into the lower chamber. After incubation for 48 h, cells were fixed and stained by 4% formaldehyde for 1 h. Then the cells were stained with crystal violet and photographed by microscope [13, 17].

### Cell oxygen consumption rate

Oxygen consumption rate was detected using Seahorse XF Cell Mito Stress Test Starter Pack according to manufacture. About 40000 cells were seeded into XF cell Culture Microplates. After 18 h, the medium was replaced with Seahorse XF DMEM (pH7.4). Oligomycin, FCCP and Rotenone were added into the plate separately according to the manufacturer’s instruction. And then it was detected by Seahorse Bioscience XF96 Extracellular Flux Analyzer. Oxygen consumption rate was normalized to cell number.

### Subcutaneous tumor mouse model

BALB/c nude mouse (5 weeks old, male) were bought from Guangzhou Qingle limited company. The research was performed in Laboratory Animal Center of Nanfang hospital (The review list of the Nanfang hospital animal ethic committee to the animal protocol, application number: NFYY-2021-1101).

The four group of cells were maintained in complete serum. When they reached their logarithmic growth, cells were collected by trypsin containing 0.25% EDTA and centrifuged at 800 rpm. Then they were resuspended by RPMI-1640 without FBS. About 300 × 10^6^ cells in 200 μl RPMI-1640 were subcutaneously injected in BALB/c nude mouse. The mice were kept in a sterile environment. On day 21 after cell injection, the mice were sacrificed, and the weight and the size of each tumor were measured. Tumor volume (mm^3^) = (*L* × *W*^2^)/2 (*L* represents the long axis and the *W* the short axis) [17].

### ATP production of tumor tissues

Every 20 mg tissue was added into 200 μl lysis buffer to be homogenized and centrifugated by 12,000 rpm, 5 min at 4 ºC. The supernatant was collected for later detection. ATP detection buffer (100 μl) diluted according to manufacture was added into 96-well plate and was opaque to light. Lysis of per group (20 μl) was added into the ATP detection buffer, and then the value was detected by luminometer.

### Statistical analysis

Data was conducted using GraphPad Prism 8. To analyze the difference in the number of adenomas between the two groups, Shapiro Wilk was used for normal distribution fitting. We detected the difference of adenoma diameter between the two groups with Kolmogorov Smirnov nonparametric test. After re-statistics, data from Fig. 4C–I was consistent with the normal distribution according to Shapiro Wilk. In the subcutaneous tumor mouse model research, the data of tumor volume and the ATP production of each group were consistent with the normal distribution according to Shapiro Wilk. Whereas, the data of tumor weight did not conform to the normal distribution according to Shapiro Wilk. However, the overall difference was statistically significant according to Kruskal Wallis test. Thus, non-parametric test of two independent samples was conducted, respectively.

Two independent t test and *one-way ANOVA* was used to determine the p value according to data sample. *Spearman’s correlation coefficient* was used to analyze the correlation of two data. *p* < 0.05 was considered statistical significant.

Additional materials and methods can be found in Supplementary Information.

## Results

### Epithelial knockout of Sirt3 promotes intestinal carcinogenesis

**Fig. 1:**
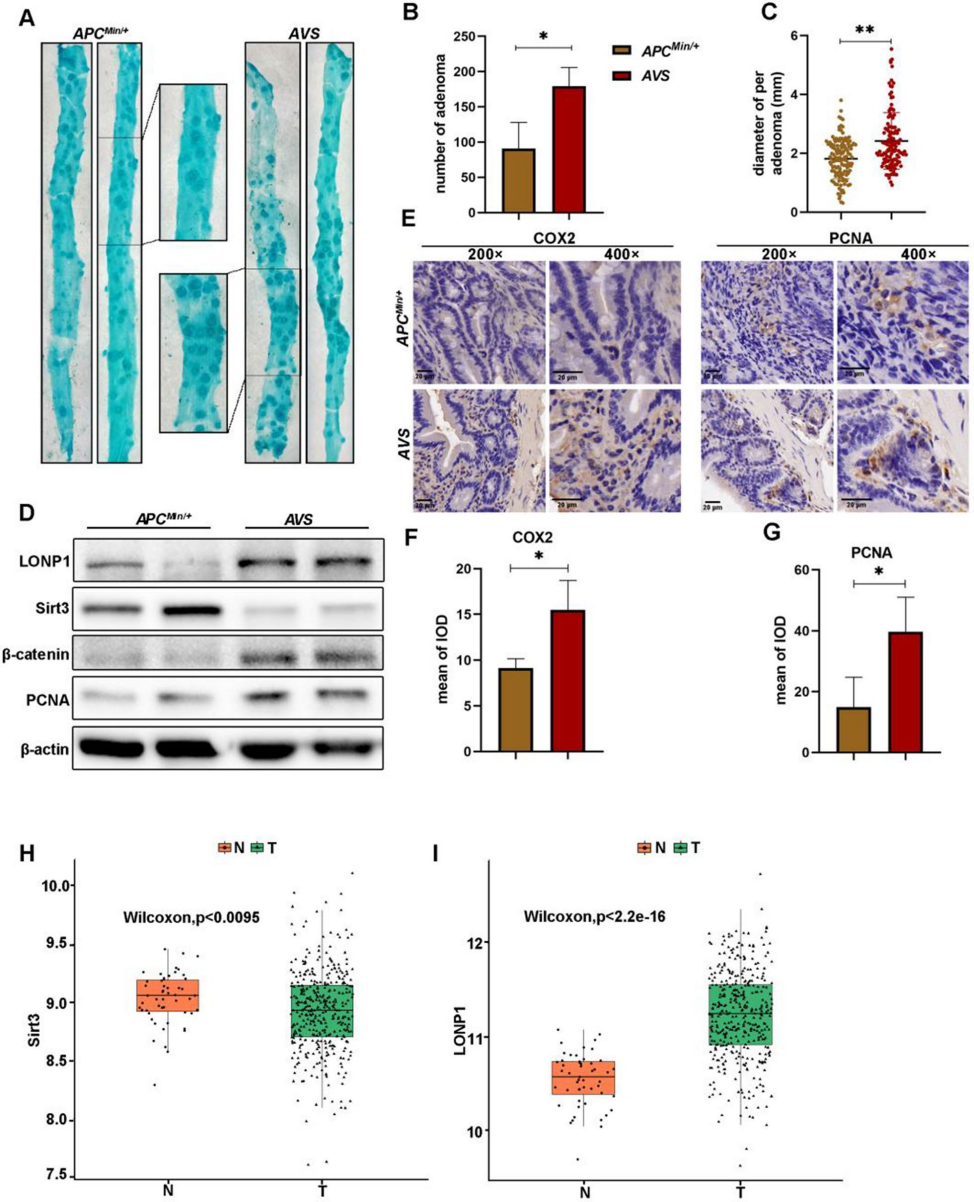
Conditional Sirt3 knockout augments adenoma number and size in *APC*^*Min/*+^ mice. **A** Tumor-bearing intestine from *AVS* (*APC*^*Min/*+^*-villin-CRE-sirt3*^*fl/fl*^) and *APC*^*Min/*+^ mice were dissected and dyed with methylene blue. **B**, **C** AVS suffer more colorectal adenoma compare to *APC*^*Min/*+^. The number and diameter of adenoma was calculated respectively. **D**, WB analysis of LONP1, Sirt3, β-catenin and PCNA expression in adenoma from *AVS* and *APC*^*Min/*+^ mice. **E**, IHC images of COX2 and PCNA expression in adenoma. **F**, **G** IHC scores showing higher expression of COX2 and PCNA in *AVS*. **H**, **I** Information analysis of expression of sirt3 and LONP1 in patient with colon cancer, respectively

To evaluate whether Sirt3 knockout (KO) can accelerate spontaneous intestinal tumorigenesis, we crossed the *Apc*^*Min/*+^ with *Villin-Cre* and *Sirt3*^*fl/fl*^ to generate *APC*^*Min/*+^*Villin-Cre Sirt3*^*fl/fl*^ mice (*AVS*) (Additional file 1: Fig. S1). The resulting *AVS* mice displayed increased adenoma number and tumor size, suggesting an exacerbated tumor burden in the intestine at 6 months (Fig. 1A–C). The multifocal neoplasmic lesions in the small intestine morphologically resembled the characteristics of familial adenomatous polyposis which is caused by germline mutation of *adenomatous polyposis coli (APC)* gene [18]. The conditional Sirt3-KO in the intestinal epithelium enhanced the expression of PCNA, β-catenin and COX-2 which are critical for intestinal carcinogenesis (Fig. 1D–G) [19]. These dysplasia were histologically in agreement with tubular adenoma or adenocarcinoma (Fig. 1E). Collectively, the loss of Sirt3 advances colorectal carcinogenesis in mice with *APC* mutation background. Interestingly, tumor tissue expressed more LONP1 protein compared to its counterpart in patient with colon cancer. Whereas, Sirt3 is the ambiguous one (Fig. 1H, I). Similar results can be obtained from The Clinical Proteomic Tumor Analysis Consortium (CPTAC) (Additional file 1: Fig. S2A–C).

### Sirt3 associates with and deacetylates LONP1

**Fig. 2:**
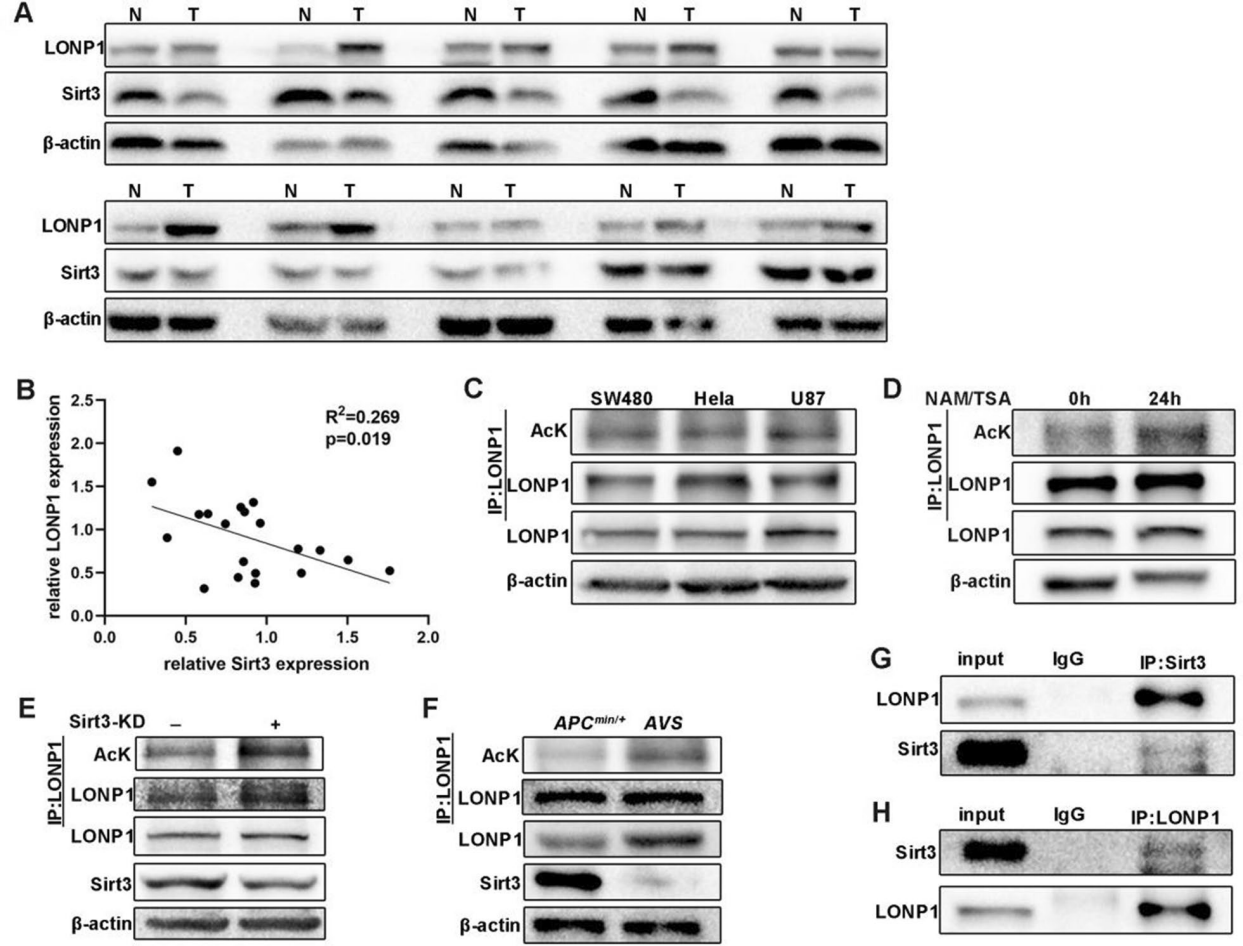
LONP1 is deacetylated by Sirt3. **A** The tumor tissue and paracancerous tissue of patient with colorectal cancer were lysated and the levels of LONP1 and Sirt3 were detected by WB. **B**, The expression of LONP1 was negatively correlated with Sirt3. R^2^ = 0.2691, p = 0.0191. **C** The acetylation of LONP1 was detected in SW480, Hela and U87 cells by WB. AcK, pan-acetyl-lysine antibody. **D**, WB showing higher level of acetylation of LONP1 after treatmen of 7.5 mM NAM and 5 mM TSA for 24 h in SW480 cells. **E** The level of acetylation of LONP1 was enhanced after stably knock-down of Sirt3 in SW480 cells. **F** Elevated LONP1 expression and its acetylation level were detected in *AVS* compared to *APC*^*Min/*+^ mice. **G**, **H** LONP1 interacts with Sirt3 in vivo

In view of the fact that LONP1 is both an oncogene [20] and a potential substrate for Sirt3 [21], their expression was probed in the specimens from CRC patients. We observed a loss of Sirt3 and a concomitant elevation of LONP1 in tumor samples. Correspondingly, we found exactly an opposite expression pattern in the para-cancerous tissues (Fig. 2A). There was a significant negative correlation between Sirt3 and LONP1 expression (Fig. 2B; Additional file 1: Fig. S3A–C). LONP1 acetylation is ubiquitously existed across SW480, Hela, AGS, U87 and HEK293T cell lines after blotting with a pan-lysine acetylation antibody (Fig. 2C; Additional file 1: Fig. S3D). We observed an elevated expression and acetylation of LONP1 both in Nicotinamide (NAM, a competitive inhibitor of the SIRT deacetylase family) treated or shRNA mediated Sirt3-KD (knockdown) SW480 and AGS cells (Fig. 2D–E, Additional file 1: Fig. S3E–F). Tissues from AVS mice further confirmed that Sirt3-KO resulted in the increment of LONP1 expression and acetylation (Fig. 2F). Sirt3 knockdown not only increased the acetylation, but also enhanced the expression of LONP1 (Fig. 2E). These results demonstrate that the loss of Sirt3 promotes acetylation of LONP1 to maintain the stabilization state of the latter.

Given that both Sirt3 and LONP1 localize in mitochondria matrix (Additional file 1: Fig. S3G) [21, 22], we therefore asked whether Sirt3 interacts with LONP1. Using anti-Sirt3 as a protein bait, we observed an association between LONP1 and Sirt3 with a conventional co-immunoprecipitation strategy. We then mutually confirmed the protein-protein interaction with anti-Sirt3 antibody and found that LONP1 can be captured by Sirt3, but not IgG. These pull-down experiments, together with a highly overlapping between Sirt3 and LONP1 (Fig. 2G–H, Additional file 1: Fig. S3H–I), indicated that Sirt3 directly interacts with LONP1. Together, Sirt3 lowers the expression of LONP1 via direct interacting with and deacetylating the latter.

### Nicotinamide inhibits Sirt3 to enhance mitochondrial LONP1 content

**Fig. 3:**
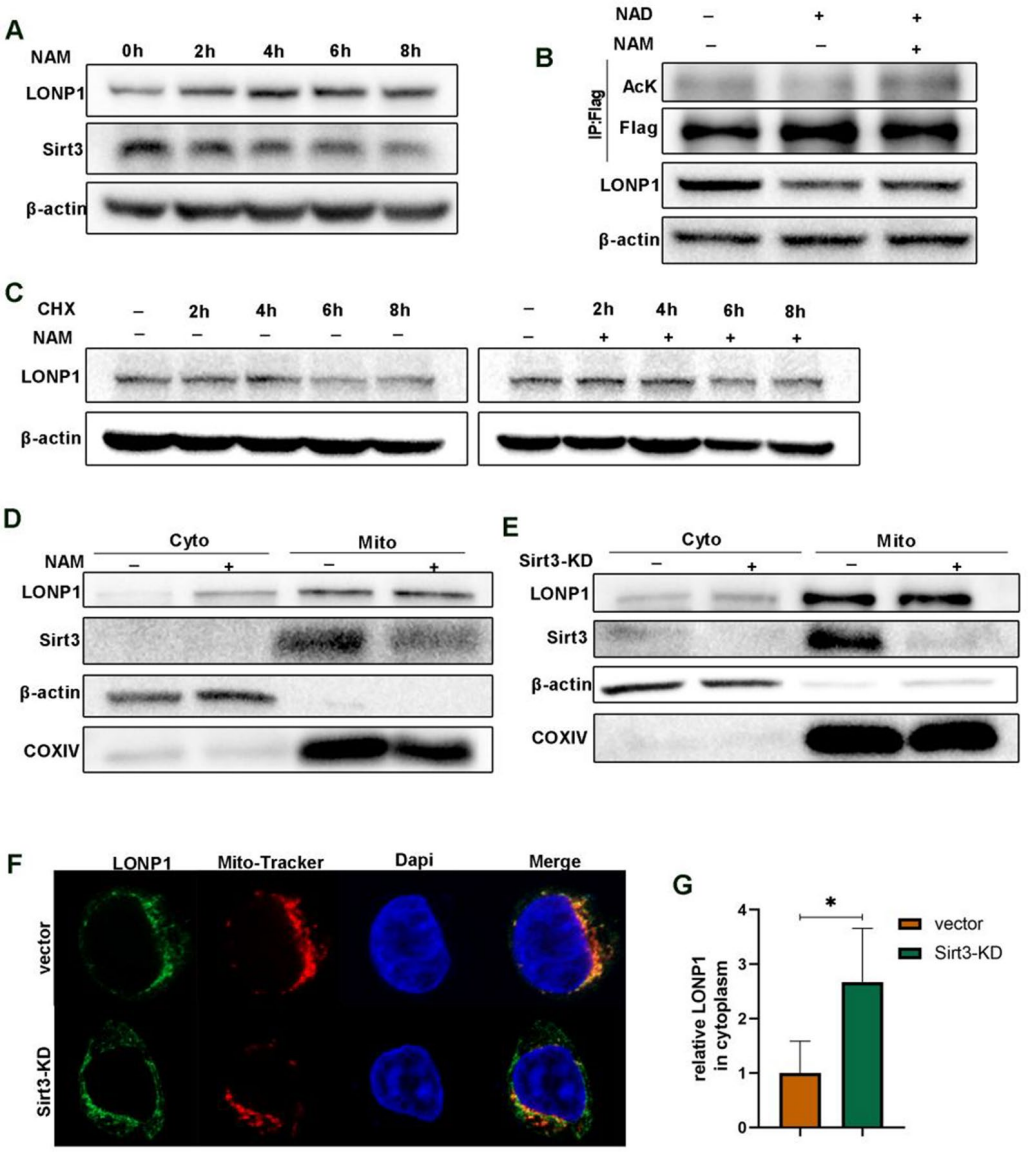
Sirt3 modulates the deacetylation and expression of LONP1. **A** The whole cells were lysed after treatment of 10 mM NAM for 0, 2, 4, 6 and 8 h respectively. WB detection of LONP1 and Sirt3 showing NAM treatment increased the expression of LONP1. **B** Sirt3 decreased the protein level of LONP1. The treatment of NAD^+^ (agonist of Sirt3) decreased the level of LONP1, while its was resisted by NAM. **C** WB detection of LONP1 after treatment of cycloheximide (75 μg ml^−1^) with of without 10 mM NAM treatment. **D**, **E** The cytoplasmic protein levels of LONP1 were increased after NAM treatment or Sirt3-knockdown. The cytoplasm and mitochondria were isolated and the protein levels of LONP1 was detected by WB in cells treated with NAM **D** and cells stably knockdown of Sirt3 **E**. **F**, **G** Fluorescence imaging revealed the increasing level of LONP1 in cytoplasm after Sirt3 deficiency **F** with confocal microscopy

Given that Sirt3 directly deacetylates LONP1, we further confirmed the conclusion by showing that NAM time-dependently dampened the expression of Sirt3 which correspondingly resulted in an increased expression of LONP1 both in SW480 and AGS cells (Fig. 3A, Additional file 1: Fig. S4A). Nicotinamide adenine dinucleotide (NAD^+^) acts as a cosubstrate to help Sirt3 deacetylating target proteins by accepting the acetyl group [23]. NAD^+^ increased the expression of Sirt3 while reduced the acetylation and expression of LONP1, which can be reversed by NAM pretreatment (Fig. 3B). To eliminate the interference of de novo protein synthesis on LONP1 abundance, SW480 cells were pre-incubated with cycloheximide, an antifungal antibiotic which specifically inhibit the cytoplasmic protein synthesis. NAM can still increase the expression of LONP1 (Fig. 3C; Additional file 1: Fig. S4B), suggested that the upregulated LONP1 protein level might be ascribe to protein degradation procedure mediated by post-translational modification, but not to the protein synthesis process.

It has been recently determined that LONP1 can also localized in the cytoplasm [24]. Our results demonstrated that LONP1 accumulated mainly in the mitochondria in Sirt3-WT (wild type) SW480 cells. However, LONP1 partially translocated to the cytosol after NAM treatment or shRNA-mediated Sirt3-KD (Fig. 3D–E, Additional file 1: Fig.S4C–D). Sirt3-KD not only enhanced the expression of mitochondrial LONP1, but also promoted the translocation of LONP1 into cytosol (Fig. 3F–G). These data suggested that NAM augments the mitochondrial expression of LONP1 through blocking Sirt3 deacetylase activity.

### Acetylation of LONP1 K145 regulates oxidative phosphorylation

**Fig. 4:**
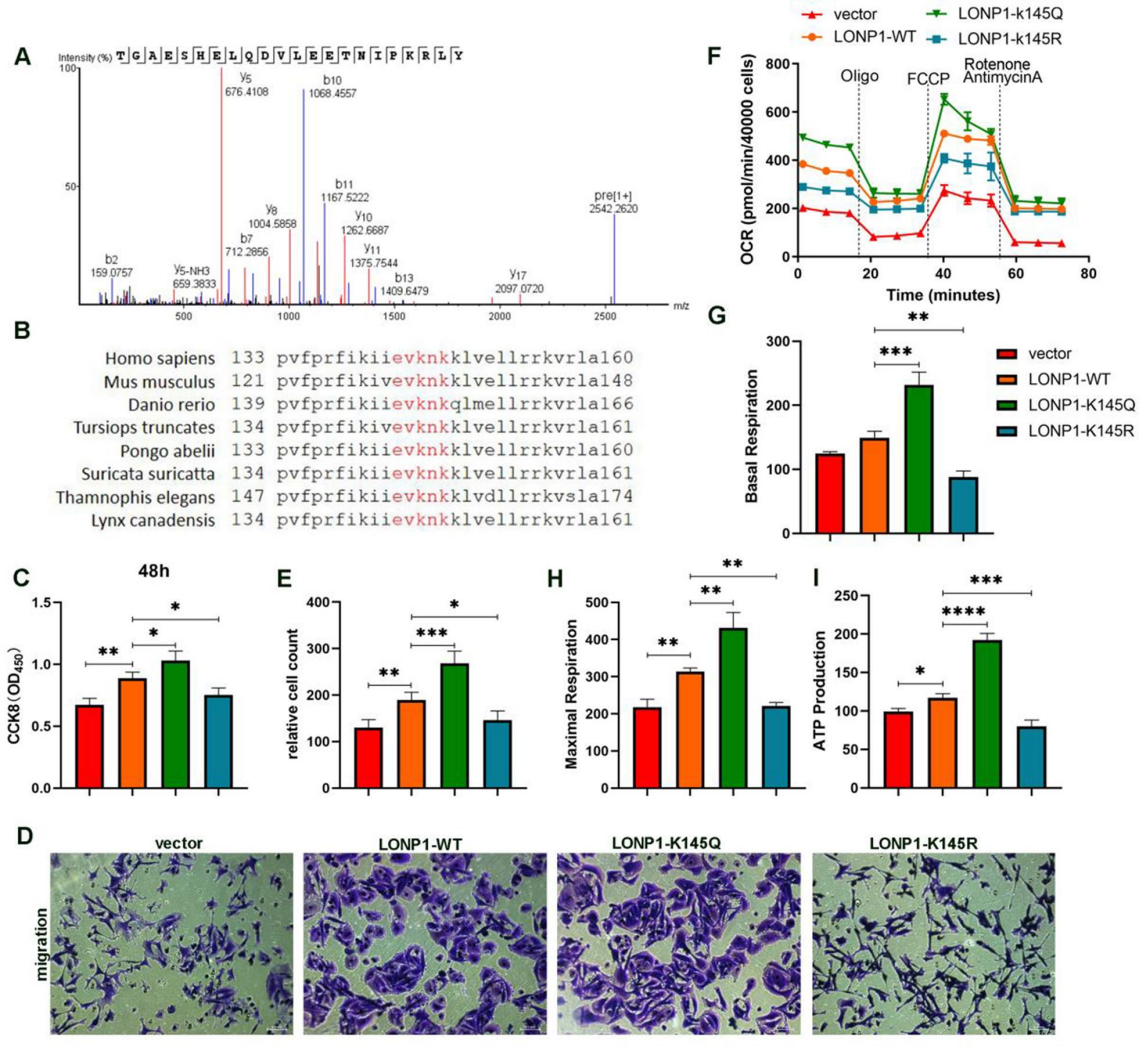
LONP1 K145 is the deacetylation site for Sirt3. **A** Mass spectrum was conducted to detect the acetylation sites of LONP1. **B** K145 of LONP1 is conserved among many species. The sequence around LONP1 K145 of several species were aligned. **C** LONP1-K145Q promotes SW480 cells growth. SW480 cells were stably transfected with vector, LONP1-WT, LONP1-K145Q and LONP1-K145R lentivirus. Cell viability of per group was detected by CCK-8 after 48 h. **D**, **E** LONP1-K145Q promotes the migration of SW480 cells. Cells were seeded in transwell. Migrated cells were counted. **F**–**I** LONP1-K145Q enhanced mitochondrial OXPHOS function. Mitochondrial function was detected by Cell Mito Stress Test kit using Seahorse analysis **F**, **G**. LONP1-K145Q enhanced the level of maximal OCR **H** and ATP production **I** of SW480 cells

Mass spectrometry demonstrated that K145 of human LONP1 was identified to be acetylated in Sirt3-KD SW480 cells (Fig. 4A). Bioinformatic analysis showed that Lys145 locates at the center of a consensus sequences “EVK^145^NK” which is highly conserved across multiple species from E. coli to Homo sapiens (Fig. 4B). To test if K145 acetylation of LONP1 is functional, we firstly generated lentivirus containing LONP1-WT and point mutations of K145R (to mimic deacetylation) and K145Q (to mimic acetylation), respectively. Next, lentivirus carrying immediate-early promoter driven LONP1-WT, K145R and K145Q infected SW480 cells were screened with puromycin to get stable overexpression cell lines. LONP1-WT overexpression heightens the proliferative and invasive ability (Fig. 4C–E). The persistent acetylation mutant K145Q elevated proliferation rate and transwell migration of SW480. On the contrary, the persistent deacetylation K145R mutant suppressed the ability of proliferation and invasion (Fig. 4C–E).

As LONP1 enhances mitochondrial functions for cancer cell survival, we developed a hypothesis that different constructs of LONP1 controls cell proliferation by modulating mitochondrial oxidative phosphorylation (OXPHOS). To verify this possibility, we assessed the mitochondrial respiratory functions among these cell lines. LONP1 K145Q increased the basal oxygen consumption rate (OCR) (Fig. 4F–G). In FCCP induced maximal respiration, there was a marked increment of OCR in LONP1 K145Q cells and a significant reduction of OCR in LONP1 K145R cells (Fig. 4F, H). Correspondingly, we found that K145Q mutation elicited an elevated ATP production while K145R mutation demonstrated defective ATP generation (Fig. 4I). These data support a mechanism that acetylation and deacetylation of LONP1 K145 in tumor cells result in opposite directions on ATP production. Collectively, the acetylation status of LONP1 advances tumor growth and invasion via promoting OXPHOS.

### Deacetylation of LONP1 at K145 enhances K63 ubiquitin binding

**Fig. 5:**
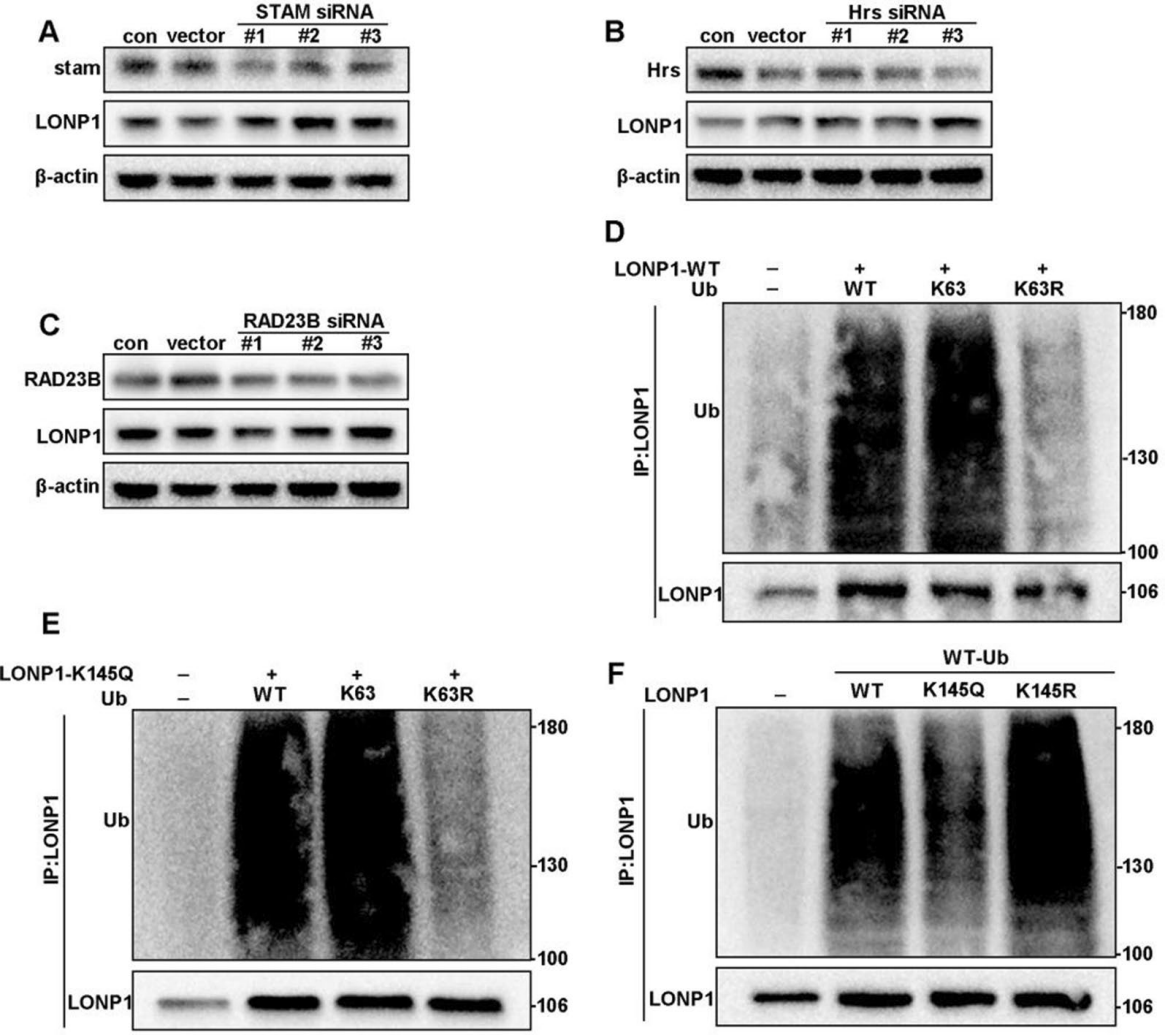
**A**–**C** STAM, Hrs and hHR23 were knocked down respectively in HEK293T cells. Then, the protein level of LONP1 was detected. **D**, **E** HA-Ub, HA-K63-Ub, HA-Ub-K63R plasmid were transfected in LONP1 WT cells and LONP1 K145Q mutation cells respectively. Cells were immunoprecipitated with LONP1 antibody followed by Western blotting. **F** HA-Ub plasmid was transfected in Flag-tagged LONP1 WT, K145Q and K145R mutation cells respectively. Ub level was detected by WB

Because protein deacetylation status potentially affect the choice of lysosomal [25] or proteasomal degradation [26], we firstly exposed AGS cells to NH_4_Cl (a lysosome inhibitor), 3-MA (an autophagy inhibitor) and MG132 (a proteasome inhibitor) and noticed that NH_4_Cl, but not MG132 (Additional file 1: Fig. S5A–C), increased the expression of LONP1, suggesting that lysosome is the major organelle responsible for LONP1 turnover. As endosomal sorting complex required for transport (ESCRT) and UV excision repair protein RAD23 homolog B (hHR23) differentially interacted with K63- or K48-tagged protein, we confirmed the above results by silencing signal transducing adapter molecule 1 (STAM) and hepatocyte growth factor-regulated tyrosine kinase substrate (Hrs) (components of ESCRT0 complex) and hHR23. As expected, both STAM and Hrs siRNA, but not hHR23 siRNA, increased the expression of LONP1 (Fig. 5A–C).

The hyperacetylation of LONP1 mutant decreased the ubiquitin binding ability, whereas the deacetylation mutant expanded the ubiquitin binding capacity. We then overexpressed wild type ubiquitin (wt-Ub), K63 ubiquiqin (K63-Ub) and K63R mutant ubiquitin in LONP1 overexpression cells and K145Q mutation cells respectively. And we found that LONP1 bound to wt-Ub and K63-Ub, but not to K63R mutant (Fig. 5D, E). Similarly, LONP1 K145R exhibited strong affinity to K63 ubiquitin, suggested that deacetylation of LONP1 K145 ensures its K63 ubiquitin binding ability (Fig. 5F). Taken together, the deacetylation of LONP1 at K145 is essential for its binding to K63 ubiquitin and subsequent degradation by lysosome system.

### LOPN1 acetylation status controls tumor growth

**Fig. 6:**
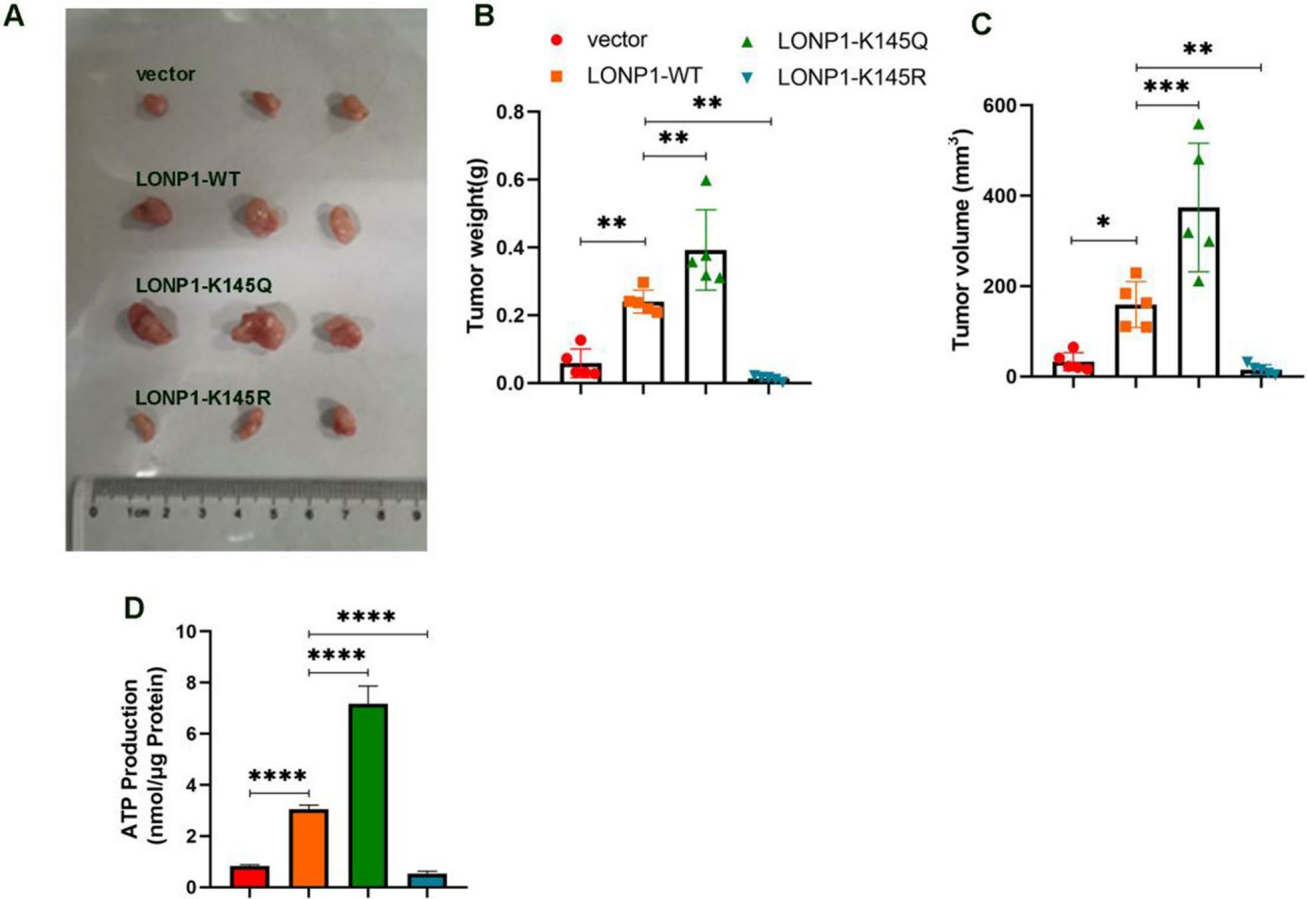
LONP1 K145 acetylation controls tumor growth. **A**–**D** K145Q mutation supported tumor growth. LONP1 WT, K145Q, K145R mutation and vector SW480 cells were subcutaneously injected into BALB/c mouse. After 21 days, the mice were sacrificed. Tumor weight and volume were measured **B**, **C**. The ATP level was detected using ATP detection kit **D**

To evaluate the influence of LONP1 acetylation on tumor growth, SW480 cells stably transfected with vector, LONP1-WT, LONP1-K145Q and LONP1-K145R were xenotransplanted to athymic nude mice (BALB/c-*nu/nu*) (Fig. 6A). LONP1-WT significantly increased the growth of tumor size and tumor weight at the end of 3 weeks. LONP1-K145Q further augmented tumor growth, whereas LONP1-K145R sharply regressed tumor progression as compared to LONP1-WT construct (Fig. 6B–C). Correspondingly, LONP1-K145Q enhance ATP production and LONP1-K145R reduce ATP content (Fig. 6D), suggested that the different construct of LONP1 governs tumor growth through regulating the energy metabolism, respectively.

**Fig. 7:**
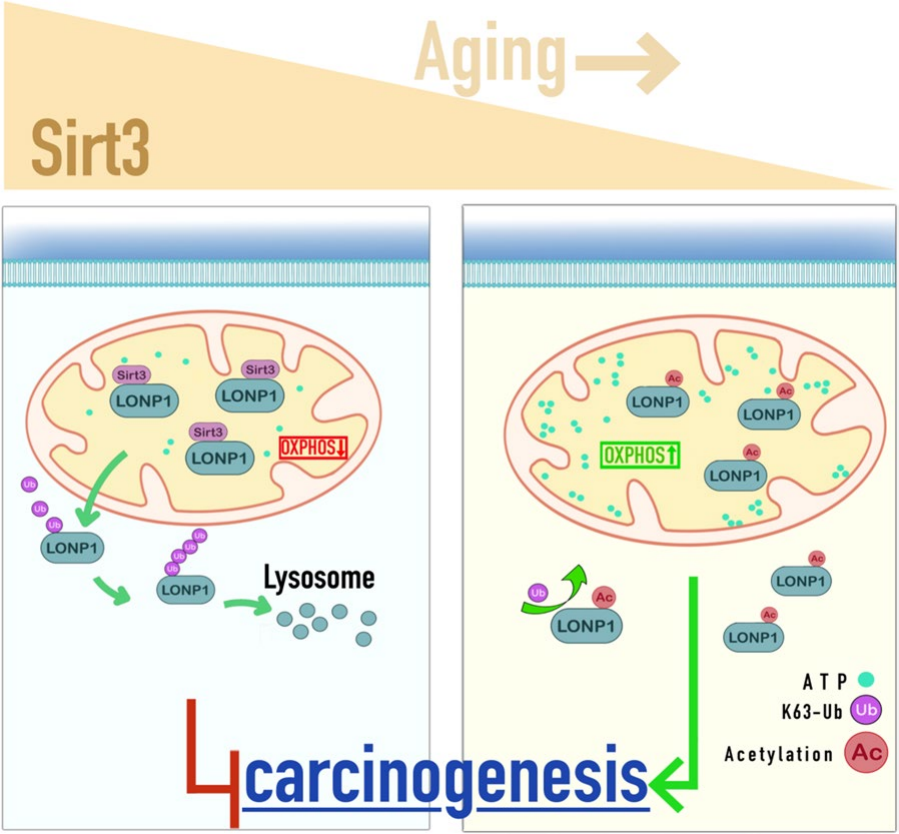
Figure abstract

Aging has been demonstrated to cause Sirt3 loss which is insufficient for the deacetylation and degradation of LONP1. The accumulated LONP1, elevated oxidative phosphorylation and glycolysis increase the risk of carcinogenesis and accelerate the growth of tumor. For the first time, we show that Sirt3 deacetylates oncogene LONP1 at K145 to attenuate ATP production and to restrict carcinogenesis (Fig. 7). In a word, Sirt3 loss during aging drives carcinogenesis by unleashing LONP1 acetylation which can be ameliorated by NAD^+^ supplementation.

## Discussion

Sirt3 is a fidelity mitochondria protein which localizes to the inner mitochondrial membrane (IMM) cristae and the matrix [27]. Fortunately, up to 90% eukaryotic LONP1 presents in the mitochondrial matrix and situates closely to the IMM [28]. The co-localization of Sirt3 and LONP1 in the mitochondria matrix is a prerequisite for their enzyme-substrate interaction [29, 30]. We identified K145 as a novel site for Sirt3 to deacetylate and to labilize LONP1. As LONP1 is an oncogene which enhances mitochondria OXPHOS and glycolysis [20, 30], our results provide a feasible solution by drawing help from Sirt3 to accelerate the degradation of LONP1.

Sirt3 has been generally recognized as a tumor suppressor which represses oxidative stress and destabilizes HIF-α [11, 31, 32]. However, there are still some different voices. Sirt3 over-expression was observed in a brain tumor family [33] and in a cohort of tongue cancer patients [34]. Also, elevated Sirt3 mRNA can be found in breast cancer patients with lymph node metastasis [35]. We carefully checked the original data and found that the number of cases included in these researches were all limited. For example, the number of control group in tongue cancer research is 8 [34] and the number of metastatic breast cancer patients is 12 [35]. Importantly, the specimen with Sirt3 over-expression were inevitably established solid tumors. Our results also showed that the expression of Sirt3 in several tumor samples from CRC patients was lower than the corresponding adjacent tissues. We therefore inferred that Sirt3 might safeguard tumorigenesis before tumor occurrence but may accelerate tumor development after tumor established [11, 36, 37]. The results that *Sirt*^*−/−*^ mice are hypersusceptible to AOM/DSS induced tumorigenesis provide solid evidence to our hypothesis [38]. To focus on epithelial ablation of Sirt3 on intestinal tumor development, we generated *AVS* mice and observed a tumorigenic phenotype which further confirmed that Sirt3 restricts tumorigenesis at the initiation stage.

LONP1 maintains a bioenergetic and biosynthetic phenotype which is required for carcinogenesis [39], which illuminates that LONP1 knockdown lowered the activities of complex I and generated less ATP to leash tumor progression [40]. In line with their results, we show that LONP1 deacetylation produces a calorie-restriction like phenotype whereas its acetylation exhibits a high ATP-yielding phenotype by utilizing K145R and K145Q to mimic hyperacetylation and hypoacetylation, respectively. The metabolic reprogramming caused by acetylation and accumulation of LONP1 represents a booster to trigger carcinogenesis in aging patients with accumulated gene mutations, such as *APC* loss or mutation [41]. Hence, the acetylation status of LONP1 may act as a rheostat to control cellular energy supply during CRC initiation.

As lysine K48 and K63 differentially guide target proteins for proteasomal or lysosomal degradation respectively, we hence deciphered it with different ubiquitin mutants and identified that lysine K63, but not K48 is required to mediate the lysosomal degradation of LONP1. Given that LONP1 knockout causes embryonic lethality in mice, we can try to control the abundance of LONP1 by facilitating K63 ubiquitination so as to produce a calorie-restriction like phenotype and also to avoid the development defects induced by genetic LONP1 ablation [42]. Together with the fact that Sirt3 interacts with and deacetylates LONP1, Sirt3 may limit the transformation procedure in cancer initiation by maintaining appropriate lysosomal degradation of LONP1 through K63-ubiquitination pathway. The loss of Sirt3 in aging might produce an endogenous oncogene accumulation phenotype to push forward the transition from minimal to increasing cancer risk between ages 40 and 50 [43]. Therefore, to maintain appropriate Sirt3 level with NAD^+^ supplementation or physical exercise may be beneficial to cancer prevention during aging [44].

Briefly, we constructed an *AVS* mouse model and demonstrated that lower expression of Sirt3 promotes the growth of intestinal adenomas. Sirt3 deacetylates LONP1 at K145, promoting its degradation through K63-UB binding pathway. LONP1 deacetylation restricts tumor cell viability by reducing the energy supply of OXPHOS which was confirmed by a subcutaneous tumor mouse model. However, our research still has some limitations. Our experiment is based on the gastrointestinal tumor cell research. Thus, our finding that the deacetylation of LONP1 by Sirt3 constrains carcinogenesis need to be confirmed in other tumor cell lines.

## Conclusion

In conclusion, we found that LONP1 K145 is a newly-identified site for Sirt3 deacetylation. LONP1 acetylation inhibits the binding of ubiquitination, leading to less protein degradation. In other words, LONP1 deacetylation inhibits its function in promoting metabolism in colorectal cancer cells and inhibits cell proliferation and tumor growth. To expand the translational significance of our research, further research is needed to explore whether exercise, calorie restriction or pharmacological agents could restrict carcinogenesis through Sirt3-LONP1 pathway.

## Supplementary Information

**Additional file 1: Figure S1.** Genotype screening of *AVS* and *APC*^*Min/+*^ mouse. A Genotype screening of *villin-CRE*. B Genotype screening of *sirt3*^*fl/fl*^*.* C Genotype screening of *APC*^*Min/+*^. **Figure S2.** The difference of Sirt3 and LONP1 abundance between colon adenocarcinoma samples and normal samples. A Proteins that differed in abundance between tumor samples and normal samples (P < 0.05). Proteins with an absolute value of log2FC greater than 0.15 were shown as gray circles, Log2FC=log2(mean protein abundance in 97 tumor samples / mean protein abundance in 100 normal samples). The red circle represented LONP1 and the green circle represented SIRT3. B Difference of LONP1 protein abundance between tumor group (T=97) and normal group (N=100), p=3.50E-19. C Difference of SIRT3 protein abundance between tumor group (T=97) and normal group (N=100), p=4.40E-05. CPTAC is a data portal containing proteome and protein modification data (such as phosphoproteome) of various cancers (PMID: 24124232). The colon adenocarcinoma proteome data (PDC Study ID: PDC000109) was downloaded at LinkedOmics (http://linkedomics.org/cptac-colon/), and was used to analyze the difference of protein abundance between tumor samples and normal samples after imputing missing expression data and normalizing expression intensities. **Figure S3.** Sirt3 interacts and deacetylates LONP1 in AGS cells. A Patient with colorectal cancer expressed more LONP1 and less Sirt3. IHC imaging showed the higher level of LONP1 and less level of Sirt3 compared to paracancerous tissue respectively. Mean of IOD of LONP1 and Sirt3 were calculated respectively (B, C). D LONP1 was acetylated in SW480, AGS and HEK293T cells. The acetylation of LONP1 was detected by WB. E NAM treatment increased the acetylation of LONP1 in AGS cells. The acetylation of LONP1 was detected by WB after treatment of NAM for 12h. F Sirt3 deficiency robustly increased the acetylation level of LONP1 in AGS cells. Sirt3 was stably knocked down in AGS cells and WB detection showing higher level of acetyaltion of LONP1. G Fluorescence confocal microscopy showing the colocalization of LONP1 and Sirt3 in AGS cells. H, I LONP1 interacts with Sirt3 in AGS cells in vivo. Data are shown as mean ± S.D., n = 3, *p < 0.05, **p < 0.01. **Figure S4.** LONP1 acetylation inhibits its degradation in AGS cells. A The AGS cells were lysated after treatmen of 10 mM NAM for 0, 2, 4, 6 and 8h respectively. WB detection of LONP1 and Sirt3 showing NAM treatment increased the expression of LONP1. B, WB detection of LONP1 after treatment of CHX (75μg ml^-1^) with of without 10 mM NAM treatment. C, D, The cytoplasmic protein levels of LONP1 were increased after NAM treatment or Sirt3-knockdown in AGS cells. The cytoplasm and mitochondria were isolated and the protein levels of LONP1 was detected by WB in cells treated with NAM (C) and cells stably knockdown of Sirt3 (D). **Figure S5.** LONP1 is degraded by lysosome and proteasome pathways. A The level of LONP1 was detected after treatment of 10mM NH_4_Cl for 0, 6, 12 and 24h respectively in AGS cells. B The level of LONP1 was detected after treatment of 2mM 3-MA for 0, 3, 6 and 9h respectively in AGS cells. C The level of LONP1 was detected after treatment of 10μM MG-132 for 0, 2, 6, 12 and 24h respectively in AGS cells.

## Data Availability

All data support the results can be found in the manuscript. Details of the data are available from the corresponding author upon reasonable request. The transcriptome data and clinical information of databases TCGA Colon and Rectal Cancer (COADREAD), GDC TCGA Colon Cancer (COAD), and GDC TCGA Rectal Cancer (READ) were obtained from UCSC Xena (https://xenabrowser.net/datapages/).

## Abbreviation

AVS: *APC* ^*Min+*^ *Villin-Cre Sirt3* ^*flfl*^ *mice*

## References

[1] DeSantis CE, Miller KD, Dale W, Mohile SG, Cohen HJ, Leach CR, Goding SA, Jemal A, Siegel RL (2019). Cancer statistics for adults aged 85 years and older, 2019. CA Cancer J Clin.

[2] Wu LE, Gomes AP, Sinclair DA (2014). Geroncogenesis: metabolic changes during aging as a driver of tumorigenesis. Cancer Cell.

[3] Carrico C, Meyer JG, He W, Gibson BW, Verdin E (2018). The mitochondrial acylome emerges: proteomics, regulation by sirtuins, and metabolic and disease implications. Cell Metab.

[4] Hebert AS, Dittenhafer-Reed KE, Yu W, Bailey DJ, Selen ES, Boersma MD, Carson JJ, Tonelli M, Balloon AJ, Higbee AJ (2013). Calorie restriction and SIRT3 trigger global reprogramming of the mitochondrial protein acetylome. Mol Cell.

[5] Joseph AM, Adhihetty PJ, Buford TW, Wohlgemuth SE, Lees HA, Nguyen LM, Aranda JM, Sandesara BD, Pahor M, Manini TM (2012). The impact of aging on mitochondrial function and biogenesis pathways in skeletal muscle of sedentary high-and low-functioning elderly individuals. Aging Cell.

[6] Amano H, Chaudhury A, Rodriguez-Aguayo C, Lu L, Akhanov V, Catic A, Popov YV, Verdin E, Johnson H, Stossi F (2019). Telomere dysfunction induces sirtuin repression that drives telomere-dependent disease. Cell Metab.

[7] Bonkowski MS, Sinclair DA (2016). Slowing ageing by design: the rise of NAD(+) and sirtuin-activating compounds. Nat Rev Mol Cell Biol.

[8] Wei Z, Song J, Wang G, Cui X, Zheng J, Tang Y, Chen X, Li J, Cui L, Liu CY, Yu W (2018). Deacetylation of serine hydroxymethyl-transferase 2 by SIRT3 promotes colorectal carcinogenesis. Nat Commun.

[9] Li M, Chiang YL, Lyssiotis CA, Teater MR, Hong JY, Shen H, Wang L, Hu J, Jing H, Chen Z (2019). Non-oncogene addiction to SIRT3 plays a critical role in lymphomagenesis. Cancer Cell.

[10] Bell EL, Emerling BM, Ricoult SJ, Guarente L (2011). SirT3 suppresses hypoxia inducible factor 1alpha and tumor growth by inhibiting mitochondrial ROS production. Oncogene.

[11] Finley LW, Carracedo A, Lee J, Souza A, Egia A, Zhang J, Teruya-Feldstein J, Moreira PI, Cardoso SM, Clish CB (2011). SIRT3 opposes reprogramming of cancer cell metabolism through HIF1alpha destabilization. Cancer Cell.

[12] Li ST, Huang Shen S, Cai Y, Xing S, Wu G, Jiang Z, Hao Y, Yuan M, Wang N (2020). Myc-mediated SDHA acetylation triggers epigenetic regulation of gene expression and tumorigenesis. Nat Metab.

[13] Zhao L, Wang H, Liu C, Liu Y, Wang X, Wang S, Sun X, Li J, Deng Y, Jiang Y, Ding Y (2010). Promotion of colorectal cancer growth and metastasis by the LIM and SH3 domain protein 1. Gut.

[14] Han Q, Xu L, Lin W, Yao X, Jiang M, Zhou R, Sun X, Zhao L (2019). Long noncoding RNA CRCMSL suppresses tumor invasive and metastasis in colorectal carcinoma through nucleocytoplasmic shuttling of HMGB2. Oncogene.

[15] Zhang F, Luo Y, Shao Z, Xu L, Liu X, Niu Y, Shi J, Sun X, Liu Y, Ding Y, Zhao L (2016). MicroRNA-187, a downstream effector of TGFbeta pathway, suppresses Smad-mediated epithelial-mesenchymal transition in colorectal cancer. Cancer Lett.

[16] Han Q, Ma Y, Wang H, Dai Y, Chen C, Liu Y, Jing L, Sun X (2018). Resibufogenin suppresses colorectal cancer growth and metastasis through RIP3-mediated necroptosis. J Transl Med.

[17] Zhang L, Zhou R, Zhang W, Yao X, Li W, Xu L, Sun X, Zhao L (2019). Cysteine-rich intestinal protein 1 suppresses apoptosis and chemosensitivity to 5-fluorouracil in colorectal cancer through ubiquitin-mediated fas degradation. J Exp Clin Cancer Res.

[18] Powell SM, Petersen GM, Krush AJ, Booker S, Jen J, Giardiello FM, Hamilton SR, Vogelstein B, Kinzler KW (1993). Molecular diagnosis of familial adenomatous polyposis. N Engl J Med.

[19] Sasaki Y, Kamei D, Ishikawa Y, Ishii T, Uematsu S, Akira S, Murakami M, Hara S (2012). Microsomal prostaglandin E synthase-1 is involved in multiple steps of colon carcinogenesis. Oncogene.

[20] Lee YG, Kim HW, Nam Y, Shin KJ, Lee YJ, Park DH, Rhee HW, Seo JK, Chae YC (2021). LONP1 and ClpP cooperatively regulate mitochondrial proteostasis for cancer cell survival. Oncogenesis.

[21] Yang W, Nagasawa K, Munch C, Xu Y, Satterstrom K, Jeong S, Hayes SD, Jedrychowski MP, Vyas FS, Zaganjor E (2016). Mitochondrial sirtuin network reveals dynamic SIRT3-dependent deacetylation in response to membrane depolarization. Cell.

[22] Gibellini L, De Gaetano A, Mandrioli M, Van Tongeren E, Bortolotti CA, Cossarizza A, Pinti M (2020). The biology of lonp1: more than a mitochondrial protease. Int Rev Cell Mol Biol.

[23] Ren T, Zhang H, Wang J, Zhu J, Jin M, Wu Y, Guo X, Ji L, Huang Q, Zhang H (2017). MCU-dependent mitochondrial Ca(2+) inhibits NAD(+)/SIRT3/SOD2 pathway to promote ROS production and metastasis of HCC cells. Oncogene.

[24] Wang YC, Chen RF, Brandacher G, Lee W, Kuo YR (2018). The suppression effect of dendritic cells maturation by adipose-derived stem cells through TGF-beta1 related pathway. Exp Cell Res.

[25] Zhao Y, Jia X, Yang X, Bai X, Lu Y, Zhu L, Cheng W, Shu M, Zhu Y, Du X (2022). Deacetylation of caveolin-1 by sirt6 induces autophagy and retards high glucose-stimulated LDL transcytosis and atherosclerosis formation. Metabolism.

[26] Wang F, Chan CH, Chen K, Guan X, Lin HK, Tong Q (2012). Deacetylation of FOXO3 by SIRT1 or SIRT2 leads to Skp2-mediated FOXO3 ubiquitination and degradation. Oncogene.

[27] Schwer B, Bunkenborg J, Verdin RO, Andersen JS, Verdin E (2006). Reversible lysine acetylation controls the activity of the mitochondrial enzyme acetyl-CoA synthetase 2. Proc Natl Acad Sci USA.

[28] Zurita RO, Shoubridge EA (2018). LONP1 Is required for maturation of a subset of mitochondrial proteins, and its loss elicits an integrated stress response. Mol Cell Biol.

[29] Liu T, Ma X, Ouyang T, Chen H, Xiao Y, Huang Y, Liu J, Xu M (2019). Efficacy of 5-aminolevulinic acid-based photodynamic therapy against keloid compromised by downregulation of SIRT1-SIRT3-SOD2-mROS dependent autophagy pathway. Redox Biol.

[30] Shin CS, Meng S, Garbis SD, Moradian A, Taylor RW, Sweredoski MJ, Lomenick B, Chan DC (2021). LONP1 and mtHSP70 cooperate to promote mitochondrial protein folding. Nat Commun.

[31] Kim HS, Patel K, Muldoon-Jacobs K, Bisht KS, Aykin-Burns N, Pennington JD, van der Meer R, Nguyen P, Savage J, Owens KM (2010). SIRT3 is a mitochondria-localized tumor suppressor required for maintenance of mitochondrial integrity and metabolism during stress. Cancer Cell.

[32] Chalkiadaki A, Guarente L (2015). The multifaceted functions of sirtuins in cancer. Nat Rev Cancer.

[33] Aury-Landas J, Bougeard G, Castel H, Hernandez-Vargas H, Drouet A, Latouche JB, Schouft MT, Ferec C, Leroux D, Lasset C (2013). Germline copy number variation of genes involved in chromatin remodelling in families suggestive of Li-Fraumeni syndrome with brain tumours. Eur J Hum Genet.

[34] Alhazzazi TY, Kamarajan P, Joo N, Huang JY, Verdin E, D'Silva NJ, Kapila YL (2011). Sirtuin-3 (SIRT3), a novel potential therapeutic target for oral cancer. Cancer Am Cancer Soc.

[35] Ashraf N, Zino S, Macintyre A, Kingsmore D, Payne AP, George WD, Shiels PG (2006). Altered sirtuin expression is associated with node-positive breast cancer. Br J Cancer.

[36] Haigis MC, Deng CX, Finley LW, Kim HS, Gius D (2012). SIRT3 is a mitochondrial tumor suppressor: a scientific tale that connects aberrant cellular ROS, the Warburg effect, and carcinogenesis. Cancer Res.

[37] Zou X, Zhu Y, Park SH, Liu G, O'Brien J, Jiang H, Gius D (2017). SIRT3-mediated dimerization of IDH2 directs cancer cell metabolism and tumor growth. Cancer Res.

[38] Zhang Y, Wang XL, Zhou M, Kang C, Lang HD, Chen MT, Hui SC, Wang B, Mi MT (2018). Crosstalk between gut microbiota and sirtuin-3 in colonic inflammation and tumorigenesis. Exp Mol Med.

[39] Gibellini L, Losi L, De Biasi S, Nasi M, Lo TD, Pecorini S, Patergnani S, Pinton P, De Gaetano A, Carnevale G (2018). LonP1 differently modulates mitochondrial function and bioenergetics of primary versus metastatic colon cancer cells. Front Oncol.

[40] Quiros PM, Espanol Y, Acin-Perez R, Rodriguez F, Barcena C, Watanabe K, Calvo E, Loureiro M, Fernandez-Garcia MS, Fueyo A (2014). ATP-dependent Lon protease controls tumor bioenergetics by reprogramming mitochondrial activity. Cell Rep.

[41] Cathcart P, Craddock C, Stebbing J (2017). Fasting: starving cancer. Lancet Oncol.

[42] Zhao K, Huang X, Zhao W, Lu B, Yang Z (2022). LONP1-mediated mitochondrial quality control safeguards metabolic shifts in heart development. Development.

[43] Dix D (2019). Human carcinogenesis: the role of age and gender. Anticancer Res.

[44] Ansari A, Rahman MS, Saha SK, Saikot FK, Deep A, Kim KH (2017). Function of the SIRT3 mitochondrial deacetylase in cellular physiology, cancer, and neurodegenerative disease. Aging Cell.

