# Supplementary material for: Sirt3 restricts tumor initiation via promoting LONP1 deacetylation and K63 ubiquitination
Source: J Transl Med. 2023 Feb 4;21:81. doi: 10.1186/s12967-023-03925-x (PMC9899405; doi:10.1186/s12967-023-03925-x)
Supplement: Supplementary file 1 — Additional file 1: Figure S1. Genotype screening of AVS and APCMin/+ mouse. A Genotype screening of villin-CRE. B Genotype screening of sirt3fl/fl. C Genotype screening of APCMin/+. Figure S2. The difference of Sirt3 and LONP1 abundance between colon adenocarcinoma samples and normal samples. A Proteins that differed in abundance between tumor samples and normal samples (P < 0.05). Proteins with an absolute value of log2FC greater than 0.15 were shown as gray circles, Log2FC=log2(mean protein abundance in 97 tumor samples / mean protein abundance in 100 normal samples). The red circle represented LONP1 and the green circle represented SIRT3. B Difference of LONP1 protein abundance between tumor group (T=97) and normal group (N=100), p=3.50E-19. C Difference of SIRT3 protein abundance between tumor group (T=97) and normal group (N=100), p=4.40E-05. CPTAC is a data portal containing proteome and protein modification data (such as phosphoproteome) of various cancers (PMID: 24124232). The colon adenocarcinoma proteome data (PDC Study ID: PDC000109) was downloaded at LinkedOmics (http://linkedomics.org/cptac-colon/), and was used to analyze the difference of protein abundance between tumor samples and normal samples after imputing missing expression data and normalizing expression intensities. Figure S3. Sirt3 interacts and deacetylates LONP1 in AGS cells. A Patient with colorectal cancer expressed more LONP1 and less Sirt3. IHC imaging showed the higher level of LONP1 and less level of Sirt3 compared to paracancerous tissue respectively. Mean of IOD of LONP1 and Sirt3 were calculated respectively (B, C). D LONP1 was acetylated in SW480, AGS and HEK293T cells. The acetylation of LONP1 was detected by WB. E NAM treatment increased the acetylation of LONP1 in AGS cells. The acetylation of LONP1 was detected by WB after treatment of NAM for 12h. F Sirt3 deficiency robustly increased the acetylation level of LONP1 in AGS cells. Sirt3 was stably knocked down in AGS cells and [file 12967_2023_3925_MOESM1_ESM.doc]

Additional file for

**Sirt3 restricts tumor initiation via promoting LONP1 deacetylation and K63 ubiquitination**

**Liyi Wu^a,1^, Qinrui Han^a,1^, Xinyi Yan^a,1^, Ye Ma^a^, Ruibo Sun^a^, Wanyu Yao^a^, Baogui Gao^a^ Qingyuan Zhang^a^, Junxiong You^a^, Hao Wang^a^, Xuegang Sun^a, b^**

∗Corresponding authors: Xuegang Sun (E-mail: sxg_smu@126.com)

1 These authors contributed equally to this work.


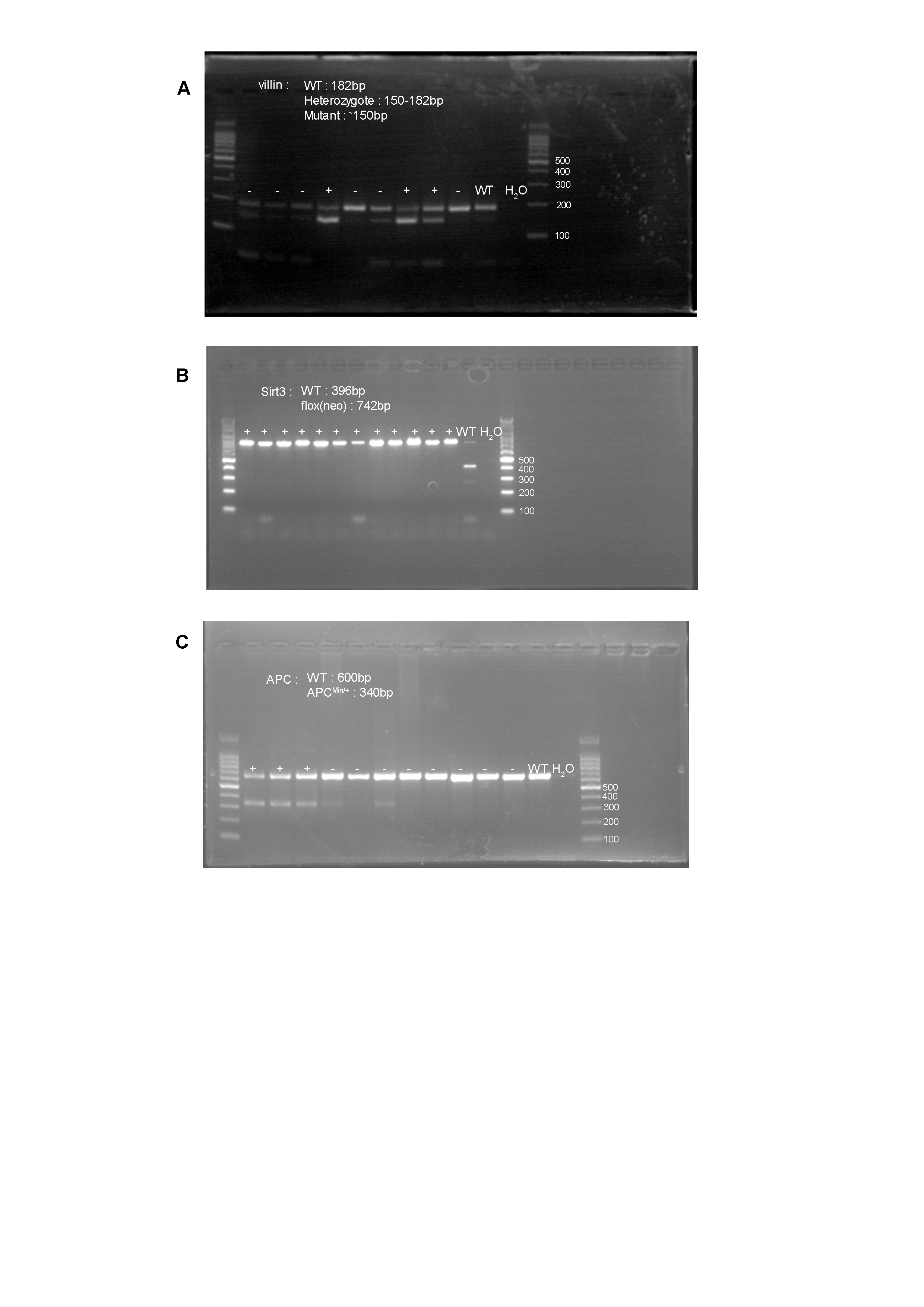


**Figure S1.** Genotype screening of *AVS* and *APC^Min/+^* mouse. A Genotype screening of *villin-CRE*. B Genotype screening of *sirt3^fl/fl^.* C Genotype screening of *APC^Min/+^*.


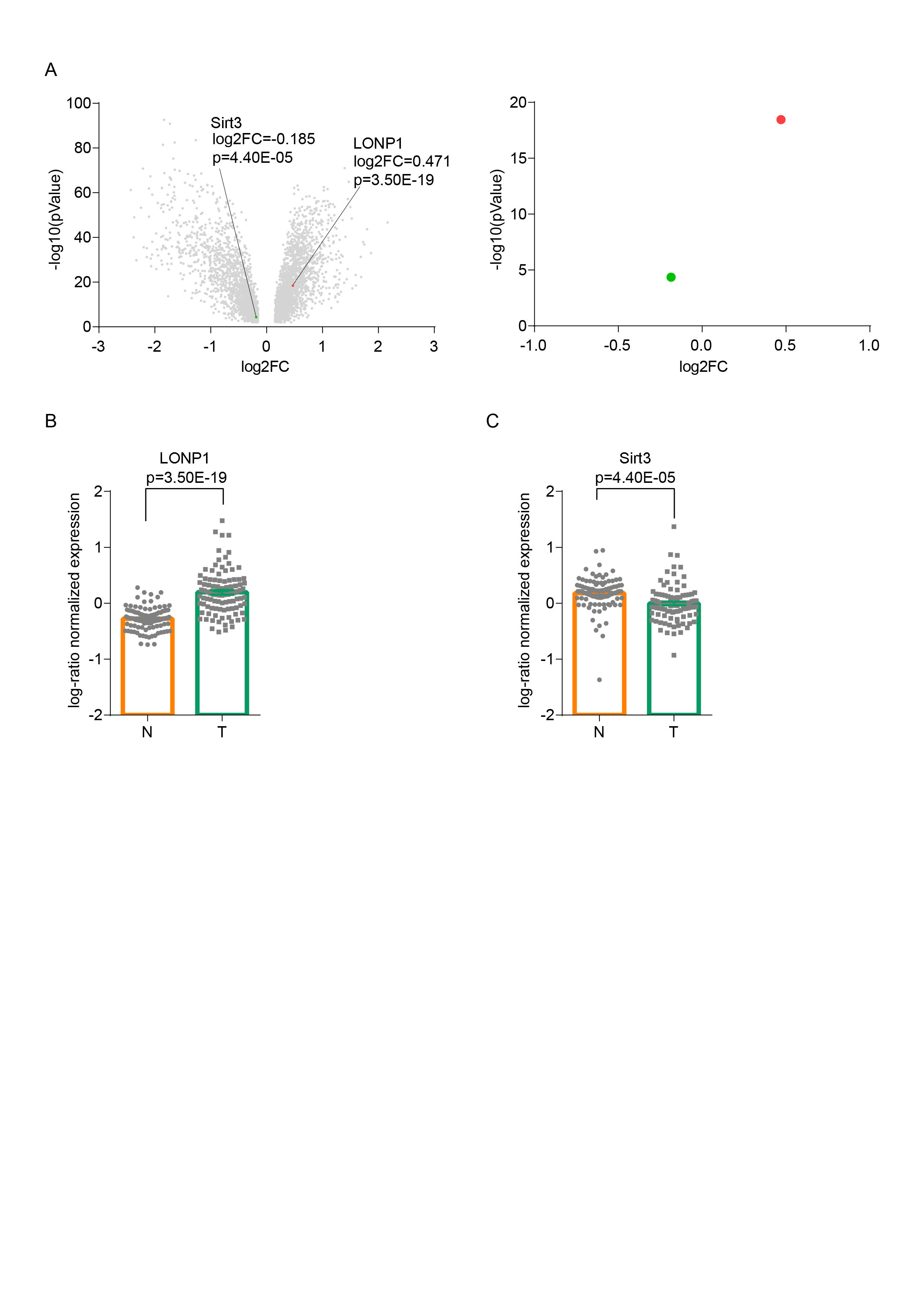


**Figure S2.** The difference of Sirt3 and LONP1 abundance between colon adenocarcinoma samples and normal samples. A Proteins that differed in abundance between tumor samples and normal samples (P < 0.05). Proteins with an absolute value of log2FC greater than 0.15 were shown as gray circles, Log2FC=log2(mean protein abundance in 97 tumor samples / mean protein abundance in 100 normal samples). The red circle represented LONP1 and the green circle represented SIRT3. B Difference of LONP1 protein abundance between tumor group (T=97) and normal group (N=100), p=3.50E-19. C Difference of SIRT3 protein abundance between tumor group (T=97) and normal group (N=100), p=4.40E-05.

CPTAC is a data portal containing proteome and protein modification data (such as phosphoproteome) of various cancers (PMID: 24124232). The colon adenocarcinoma proteome data (PDC Study ID: PDC000109) was downloaded at LinkedOmics (http://linkedomics.org/cptac-colon/), and was used to analyze the difference of protein abundance between tumor samples and normal samples after imputing missing expression data and normalizing expression intensities.


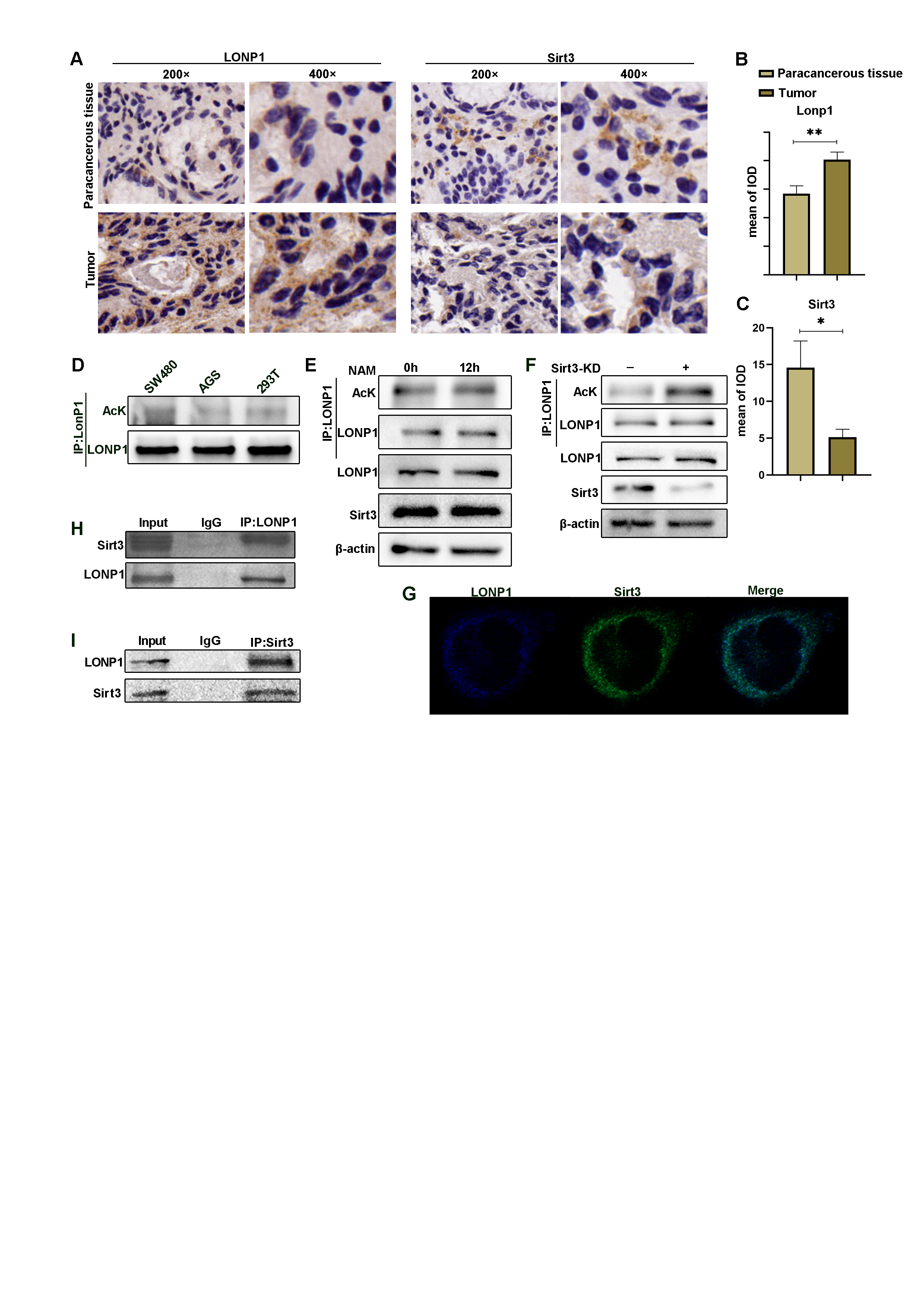


**Figure S3.** Sirt3 interacts and deacetylates LONP1 in AGS cells. A Patient with colorectal cancer expressed more LONP1 and less Sirt3. IHC imaging showed the higher level of LONP1 and less level of Sirt3 compared to paracancerous tissue respectively. Mean of IOD of LONP1 and Sirt3 were calculated respectively (B, C). D LONP1 was acetylated in SW480, AGS and HEK293T cells. The acetylation of LONP1 was detected by WB. E NAM treatment increased the acetylation of LONP1 in AGS cells. The acetylation of LONP1 was detected by WB after treatment of NAM for 12h. F Sirt3 deficiency robustly increased the acetylation level of LONP1 in AGS cells. Sirt3 was stably knocked down in AGS cells and WB detection showing higher level of acetyaltion of LONP1. G Fluorescence confocal microscopy showing the colocalization of LONP1 and Sirt3 in AGS cells. H, I LONP1 interacts with Sirt3 in AGS cells in vivo. Data are shown as mean ± S.D., n = 3, **p* < 0.05, ***p* < 0.01.


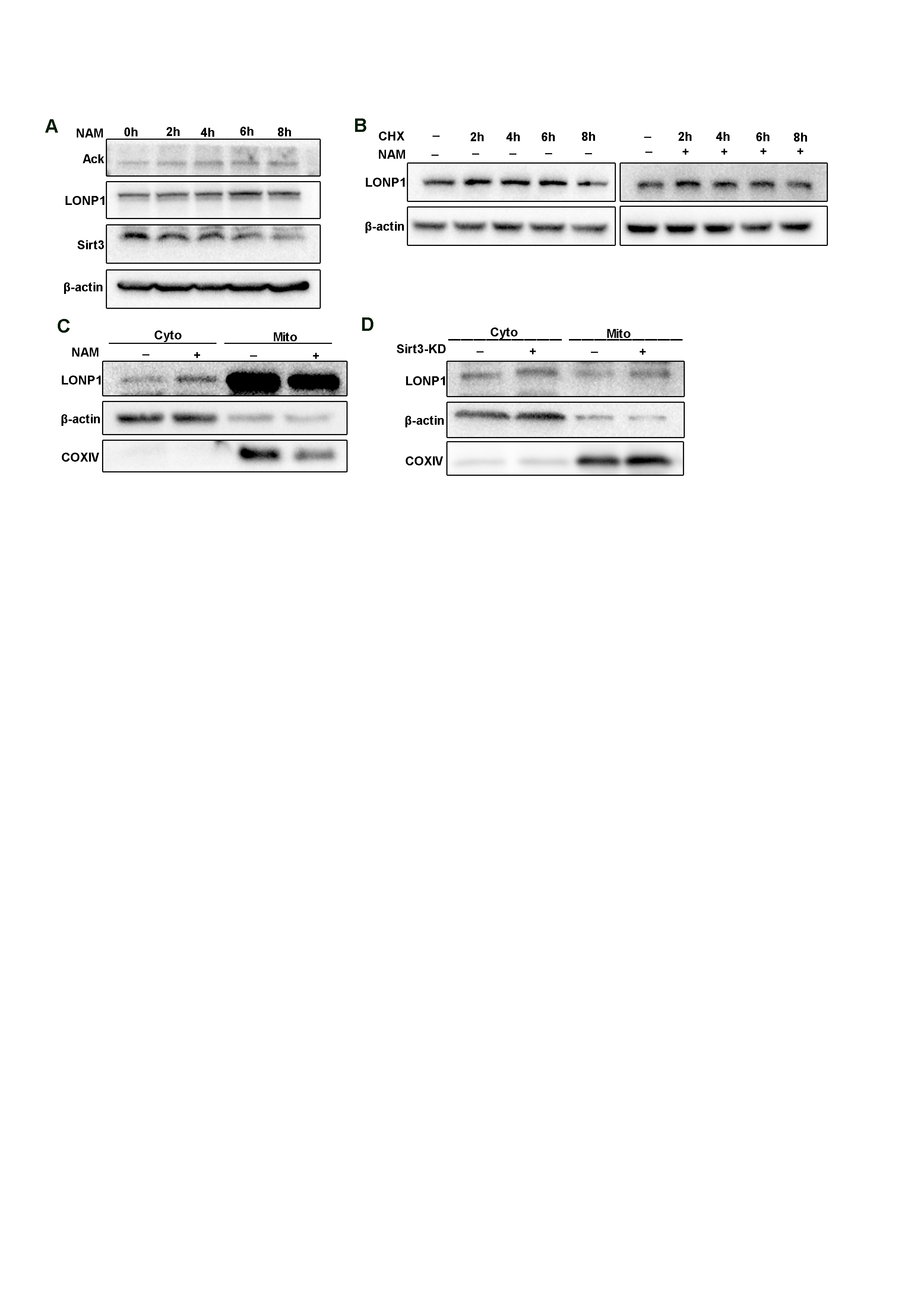


**Figure S4.** LONP1 acetylation inhibits its degradation in AGS cells. A The AGS cells were lysated after treatmen of 10 mM NAM for 0, 2, 4, 6 and 8h respectively. WB detection of LONP1 and Sirt3 showing NAM treatment increased the expression of LONP1. B, WB detection of LONP1 after treatment of CHX (75μg ml^-1^) with of without 10 mM NAM treatment. C, D, The cytoplasmic protein levels of LONP1 were increased after NAM treatment or Sirt3-knockdown in AGS cells. The cytoplasm and mitochondria were isolated and the protein levels of LONP1 was detected by WB in cells treated with NAM (C) and cells stably knockdown of Sirt3 (D).


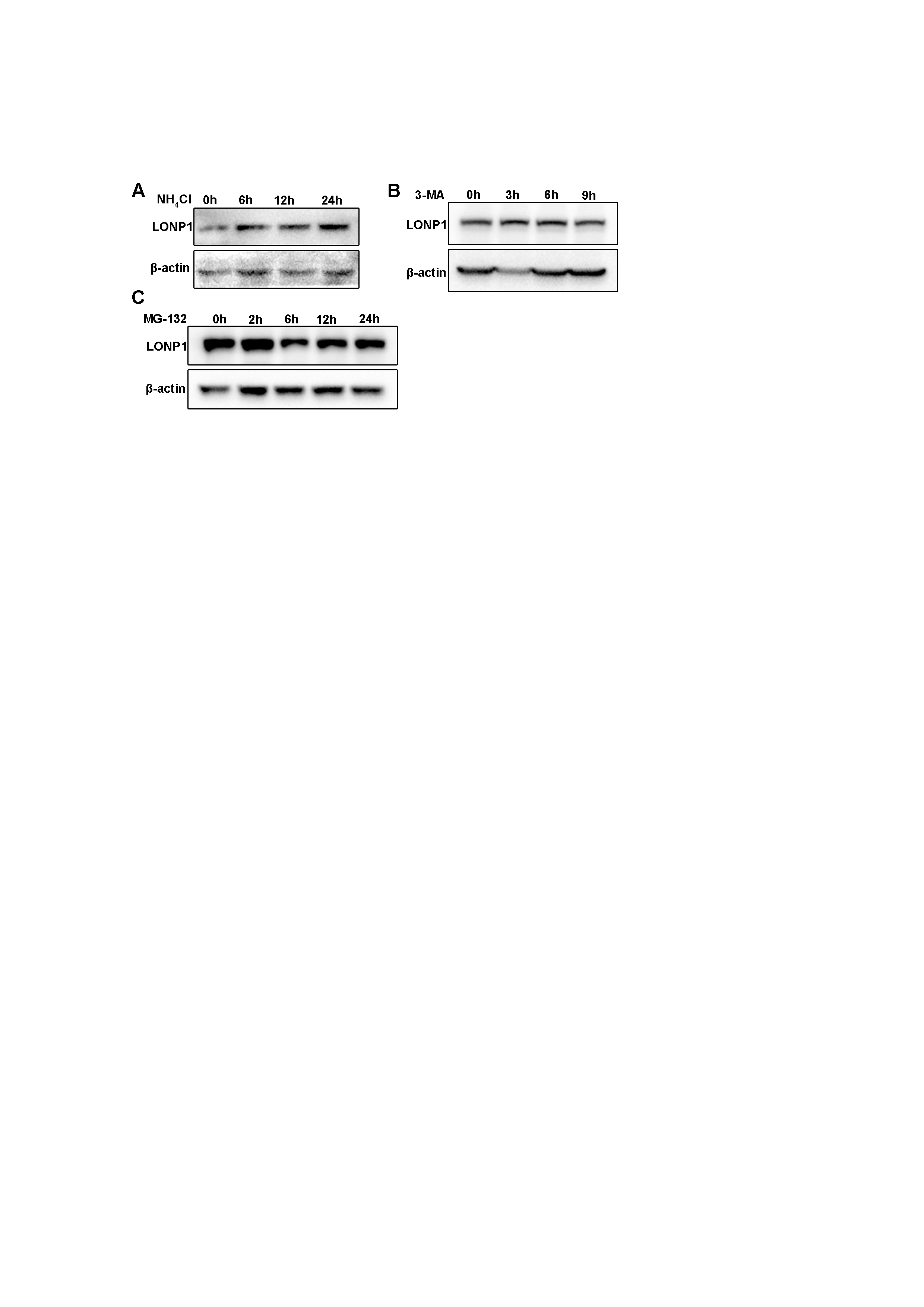


**Figure S5.** LONP1 is degraded by lysosome and proteasome pathways. A The level of LONP1 was detected after treatment of 10mM NH_4_Cl for 0, 6, 12 and 24h respectively in AGS cells. B The level of LONP1 was detected after treatment of 2mM 3-MA for 0, 3, 6 and 9h respectively in AGS cells. C The level of LONP1 was detected after treatment of 10μM MG-132 for 0, 2, 6, 12 and 24h respectively in AGS cells.
